# A Quantitative Look at Fluorosis, Fluoride Exposure, and Intake in Children Using a Health Risk Assessment Approach

**DOI:** 10.1289/ehp.7077

**Published:** 2004-09-14

**Authors:** Serap Erdal, Susan N. Buchanan

**Affiliations:** ^1^Division of Environmental and Occupational Health Sciences, School of Public Health, and; ^2^Departments of Occupational Medicine and Family Medicine, College of Medicine, University of Illinois at Chicago, Chicago, Illinois, USA

**Keywords:** children, exposure, fluoride, multi-pathway, risk

## Abstract

The prevalence of dental fluorosis in the United States has increased during the last 30 years. In this study, we used a mathematical model commonly employed by the U.S. Environmental Protection Agency to estimate average daily intake of fluoride via all applicable exposure pathways contributing to fluorosis risk for infants and children living in hypothetical fluoridated and non-fluoridated communities. We also estimated hazard quotients for each exposure pathway and hazard indices for exposure conditions representative of central tendency exposure (CTE) and reasonable maximum exposure (RME) conditions. The exposure pathways considered were uptake of fluoride via fluoridated drinking water, beverages, cow’s milk, foods, and fluoride supplements for both age groups. Additionally, consumption of infant formula for infants and inadvertent swallowing of toothpaste while brushing and incidental ingestion of soil for children were also considered. The cumulative daily fluoride intake in fluoridated areas was estimated as 0.20 and 0.11 mg/kg-day for RME and CTE scenarios, respectively, for infants. On the other hand, the RME and CTE estimates for children were 0.23 and 0.06 mg/kg-day, respectively. In areas where municipal water is not fluoridated, our RME and CTE estimates for cumulative daily average intake were, respectively, 0.11 and 0.08 mg/kg-day for infants and 0.21 and 0.06 mg/kg-day for children. Our theoretical estimates are in good agreement with measurement-based estimates reported in the literature. Although CTE estimates were within the optimum range for dental caries prevention, the RME estimates were above the upper tolerable intake limit. This suggests that some children may be at risk for fluorosis.

Nearly two-thirds of the U.S. population receives drinking water from municipalities that add fluoride to their water systems to prevent dental caries [Centers for Disease Control and Prevention (CDC) 2002]. The CDC hails fluoridation of drinking water as one of the 10 great public health achievements of the 20th century (CDC 1999). The first Surgeon General’s report on oral health in the United States credits fluoridation for dramatically lowering caries rates. Several studies have shown caries reduction of up to 60% after fluoridation [U.S. Department of Health and Human Services (DHHS) 2000a].

Although the efficacy of drinking-water fluoridation is well accepted by the scientific community and policy makers, the benefits are not without consequence. Ingestion of fluoride during the formative years of a child’s enamel development can cause dental fluorosis—a condition marked by permanent, often pronounced staining of adult teeth. Reports of fluorosis prevalence in North American children range widely depending on public water fluoridation status (Clark 1994; Mascarenhas 2000; Riordan and Banks 1991; Tabari et al. 2000). In the National Survey of Dental Caries in U.S. school children (1986–1987), 22% of children examined had fluorosis (Brunelle 1989). In 1998, 69% of children 7–11 years of age examined in a suburban Boston pediatric practice were found to have fluorosis (Morgan et al. 1998). Children from a fluoridated community in North Carolina showed a prevalence of 78% with fluorosis (Lalumandier and Rozier 1995). In nonfluoridated communities, fluorosis prevalence reported in a number of studies conducted during 1990–2000 ranged from 3 to 45% (Clark 1994; Mascarenhas 2000; Riordan and Banks 1991; Tabari et al. 2000).

Several studies point to other sources of fluoride besides fluoridated drinking water (e.g., fluoride toothpaste, fluoride supplements, infant formula and beverages produced with fluoridated water, food grown in soil containing fluoride or irrigated with fluoridated water, and cow’s milk from livestock raised on fluoride-containing water and feed, and soil) that contribute to overall fluoride intake and therefore may contribute to dental fluorosis (Fomon et al. 2000; Jackson et al. 2002; Levy 1994; Levy et al. 2001; Pendrys and Stamm 1990). In this study, we evaluated total fluoride intake and fluorosis risk of infants and children using quantitative health risk assessment. Although several published studies in the past decade have measured daily intake rates of fluoride from various sources such as diet, toothpaste, and infant formula (Fomon et al. 2000; Jackson et al. 2002; Levy 1994; Levy et al. 2001; Pendrys and Stamm 1990), none has systematically considered cumulative fluoride intake from all significant sources combined. We performed a comparative analysis of fluoride intake in fluoridated and nonfluoridated communities by characterizing the exposures via all significant exposure pathways applicable for infants and children in two age groups: infants less than 1 year of age and children 3–5 years of age. The analysis was limited to formula-fed infants only.

## Materials and Methods

We used the risk assessment paradigm developed by the National Academy of Sciences (1983), which is commonly used by federal environmental agencies in the United States to inform decisions regarding risk priorities, risk ranking, and health-based environmental standard development [U.S. Environmental Protection Agency (EPA) 1989, 1995]. This risk assessment model, in general, consists of the following four steps: hazard identification, dose–response assessment, exposure assessment, and risk characterization. We applied this four-step risk assessment paradigm to quantitatively estimate exposure-pathway–specific and cumulative daily average intake of fluoride by infants and children. The significance of dental fluorosis has been controversial at times, and there are differing perspectives by differing agencies and organizations charged with protecting the public health. Although the U.S. EPA considers fluorosis a cosmetic effect rather than an adverse health effect, the World Health Organization (WHO) treats fluorosis as an adverse health effect affecting millions of people around the world (WHO 2001, 2002). In this study, we estimated cumulative daily dose and health risk to determine the exposure pathways and conditions resulting in increased likelihood for dental fluorosis in children, with the vision that such information would be beneficial in identifying exposure pathways of concern and in managing risks for fluorosis. Therefore, application of a quantitative risk assessment model is appropriate for determining acceptability of risks associated with exposure to any chemical, whether or not that chemical has a specific adverse health effect.

### Hazard identification.

Several health effects are associated with fluoride ingestion, ranging from nausea to neurotoxic effects to death (Mullins et al. 1998; Vogt et al. 1982). The effect of concern in our risk assessment is the most common effect of chronic ingestion of fluoride in the form of fluoride salts: dental fluorosis. Fluorosis occurs as permanent teeth are forming and is characterized by permanent hypomineralization. It appears initially as white streaks or mottling on the tooth enamel. With continued systemic exposure to fluoride, these streaks become white patches, progressing to brown stains and pitting. The exact age at which teeth are most vulnerable is somewhat controversial, with opinions ranging from the prenatal stage of permanent tooth formation to 3–6 years of age, when maximal mineralization occurs. It is generally accepted, however, that after 6–8 years of age, teeth are no longer susceptible to the adverse effects of fluoride.

### Dose–response assessment.

The U.S. EPA publishes a database of toxicity values derived from dose–response relationships relating exposure (dose) to health effect for various chemicals found in the environment. This database, called the Integrated Risk Information System (IRIS; U.S. EPA 2003), provides the toxicity values [e.g., reference dose (RfD)] for individual non-carcinogenic chemicals. The RfD is an estimate of the daily exposure to children and adults that is likely to be without appreciable risk of deleterious effects during a lifetime, with uncertainty spanning perhaps an order of magnitude. The RfD published by the EPA for fluoride is 0.06 mg/kg-day and is based on the no observed adverse effects level (NOAEL) of 0.06 mg/kg-day and uncertainty and modifying factors of unity (U.S. EPA 2003). Uncertainty factors were not deemed necessary because NOAEL was derived from a chronic study focusing on the critical effect (dental fluorosis) in a sensitive population of humans (children). The scientific basis and rationale of the fluoride RfD (Burt 1992; Hodge 1950) can be found in IRIS and is beyond the scope of this article.

### Exposure assessment.

The populations of interest, the pathways by which exposure may occur, and the magnitude, frequency, and duration of these potential exposures are identified in this step. The population of interest in this analysis is infants (< 1 year of age) and children (3–5 years of age). An estimated daily intake (EDI) is calculated for each exposure pathway using a number of exposure parameters using Equation 1 (U.S. EPA 1992):


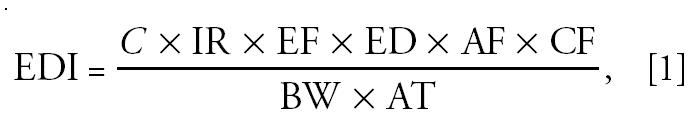


where EDI is the estimated daily intake (milligrams per kilogram per day), *C* is the concentration in a specific medium (milligrams per liter or milligrams per kilogram), IR is the ingestion or intake rate (milligrams per day), EF is the exposure frequency (days per year), ED is the exposure duration (years), AF is the absorption factor (unitless), CF is the conversion factor (10^−6^ kg/mg), BW is the body weight (kilograms), and AT is the averaging time (days).

The exposure pathways considered are as follows: pathway A, ingestion of fluoridated public drinking water; B, ingestion of soft drinks and fruit juices (beverages); C, consumption of infant formula; D, ingestion of cow’s milk; E, consumption of foods; F, incidental ingestion of soil; G, ingestion of fluoride supplement tablets; and H, incidental ingestion of fluoride toothpaste. The exposure pathways A–E and G are included in the estimation of cumulative fluoride intake for infants. All exposure pathways except pathway C (infant formula) are included to estimate cumulative fluoride intake of children.

Using Equation 1, the EDI for each exposure pathway is calculated by identifying appropriate values for exposure parameters (e.g., concentration, ingestion rate, body weight, exposure frequency, exposure duration) for the two age groups. Two values for each exposure parameter are used in characterizing potential exposures: one value to represent an average or central tendency exposure (CTE) and another value for the high-end or reasonable maximum exposure (RME), which is intended to represent a plausible worst-case exposure (U.S. EPA 1989). The RME estimates are often used by the EPA when making regulatory decisions and recommendations regarding acceptability of health risk to humans.

Age-specific values used in the calculation for EDI (Equation 1) can be found in Table 1. We consulted the U.S. EPA (2002) for the estimation of average daily fluoride intake via all the exposure pathways except consumption of infant formula (pathway C), ingestion of fluoride supplements (pathway G), and incidental ingestion of toothpaste (pathway H). A more in-depth discussion about the rationale for exposure-pathway–specific exposure parameters shown in Table 1—specifically, fluoride concentrations in each exposure medium—is presented below. Exposure frequency was assumed to be 365 days per year. Exposure duration was 1 year for infants and 2 years for children. Exceptions to these for EF and ED variables in Equation 1 are also noted below. The AT is equal to ED times 365 days/year. We used average body weight of 8.4 kg for 2- to 12-month-old male and female infants, respectively, in the U.S. population, based on survey data from 1988 to 1994 (U.S. EPA 2002), as the body weight of infants. Using the same data source, we estimated the mean body weight for the 3- to 5-year-old group in a similar fashion at 17.2 kg. The estimation of EDI also requires information on absorption (or bioavailability) factor. Fluoride is readily absorbed from the gastrointestinal tract, with estimates of absorption ranging from 75 to 100% [Agency for Toxic Substances and Disease Registry (ATSDR) 2001; Ekstrand and Ehrnebo 1980]. In toothpaste, sodium fluoride is 100% available as fluoride ion (Alhaique et al. 1982), and studies show a linear relationship between amount of tooth-paste ingested and serum levels of fluoride (Ekstrand and Ehrnebo 1980). Therefore, the AF in Equation 1 is assumed to be unity.

### Pathway A: ingestion of drinking water.

The U.S. Public Health Service sets optimal drinking water fluoride levels based on geographic temperature bands and corresponding water consumption rates (Lalumandier and Jones 1999). The recommended fluoride concentration in temperate zones is 1 ppm, or 1 mg/L. A recent study conducted by the U.S. Department of Agriculture (USDA) that measured fluoride content of nationally representative municipal water samples from 24 consolidated metropolitan statistical areas in the United States revealed that either water is fluoridated and contains approximately 1 mg/L of fluoride or it is not fluoridated with undetectable fluoride concentration (Miller-Ihli et al. 2003). The USDA study found that approximately 40% of the water samples were fluoridated with a mean concentration of 1.01 ± 0.15 mg/L. We assumed that the water in non-fluoridated areas does not contain any fluoride.

The use of bottled water as the primary source of drinking water has increased in the United States. The American Dental Association (ADA) recently called for labeling of fluoride concentrations on bottled water because of increased use of bottled water not only as a drinking water source but also in preparation of infant formulas and various foods. Bartels et al. (2000) examined fluoride concentrations of five commercially available bottled water products. The results indicated that although there were significant differences in fluoride concentrations among different brands and between different batches from the same brand, all products had fluoride concentrations lower than the ADA-accepted standard for optimally fluoridated water (i.e., 0.7–1.2 mg/L dependent on the average maximum daily air temperature of an area). Because widespread use of bottled water is a recent phenomenon, there are limited data to ascertain how bottled water intake is affecting children’s teeth. Our intake/risk estimates for infants and children consuming non-fluoridated tap water would most likely be equivalent to intake/risk estimates of those consuming bottled water as their drinking water source because most bottled water in the United States is currently not fluoridated. However, this may change in the future because of pressure from consumer groups and federal/state regulatory agencies.

### Pathway B: ingestion of soft drinks and fruit juices.

The ingestion of soft drinks and commercially prepared fruit juices has more than doubled in the last 25 years (Levy 1994). Because these beverages are usually prepared with fluoridated water, they can be a significant source of fluoride. Pang et al. (1992) reported the fluoride content of sodas, juices, punches, tea, and Gatorade purchased in North Carolina. Fluoride levels were highly variable, ranging from < 0.1 to 6.7 mg/L. We used the weighted average of these reported concentrations (0.76 mg/L) in calculating the EDI for this pathway.

### Pathway C: consumption of infant formula.

Infant formula processed with fluoridated water may be a significant source of fluoride in infants. In 1979, because of the concern about fluoride intake in infants, formula manufacturers voluntarily agreed to lower the concentration of fluoride in their products (Fomon et al. 2000; Levy 1994). However, fluoridated water used to reconstitute or dilute powdered or concentrated preparations remains a concern. An average concentration of 0.65 mg/kg fluoride is used in the EDI calculation. This concentration was derived based on a survey of fluoride concentrations in ready-to-use formula (mean, 0.23 mg/kg fluoride), concentrated liquid (mean, 0.6 mg/kg fluoride), and powdered concentrate (1.13 mg/kg fluoride) sold in retail stores in the United States (ATSDR 2001; Dabeka and McKenzie 1987). The intake rate of infant formula was estimated from feeding recommendations by Behrman and Vaughn (2000).

### Pathway D: ingestion of cow’s milk.

Cows ingesting fluoridated water or feed processed with fluoridated water produce milk containing fluoride. The mean fluoride concentration of 0.041 mg/kg (range, 0.007–0.086 mg/kg) reported in a Canadian study (Dabeka and McKenzie 1987) that surveyed fluoride concentrations in 68 samples of milk sold in retail stores across Canadian provinces was used in this analysis.

Human breast milk contains very low levels of fluoride (0.004 mg/L in nonfluoridated and 0.01 mg/L in fluoridated areas) even when intake by the mother is high (Fomon et al. 2000; Levy 1994). Moreover, the percentage of exclusively breast-fed infants at 6 months of age in the United States was only 22% in 1995 (Ryan 1997). For these reasons, only formula-fed infants are included in this analysis. Exclusively breast-fed infants will have a much lower average daily fluoride intake for the duration of the breast-feeding period than will formula-fed infants.

### Pathway E: consumption of food.

Dabeka and McKenzie (1995) determined fluoride concentrations in individual food items and food composites in various categories (milk and dairy products, meat and poultry, soups, bakery goods and cereals, vegetables, fruits and fruit juices, fats and oils, sugar and candies, beverages, and other miscellaneous items) purchased in Winnipeg, Canada. Food categories with the highest mean fluoride levels were fish (2.118 mg/kg), soups (0.606 mg/kg), and beverages (1.148 mg/kg). The mean fluoride concentration in all samples, including milk, various beverages and fruit juices, and tap water, was 0.325 mg/kg, ranging from 0.011 to 4.970 mg/kg. Using these data, we estimated the mean fluoride concentration of 0.262 and 0.29 mg/kg fluoride in foods potentially consumed by infants and children, respectively. This estimate does not include milk, beverages and fruit juices, and tap water because these are treated separately in our analysis. For infants, certain food items were excluded from their diet (e.g., cold cuts, lunch meat, cured meats, honey), and fluoride exposure due to food consumption was limited to 8 months, starting at 4 months of age.

### Pathway F: incidental ingestion of soil.

Children inadvertently ingest soil through normal hand-to-mouth behavior. Industrial sites, hazardous waste sites containing fluoride, and soil contaminated with phosphate-containing fertilizers may have higher levels of fluoride. We used the mean fluoride concentration in soils and other surface materials in the United States (430 mg/kg; range, 10–37,000 mg/kg) (ATSDR 2001) in the calculation of the EDI for the incidental soil ingestion pathway. Because children < 1 year old are not ambulatory, the average daily fluoride intake for this pathway is calculated for children 3–5 years of age only.

### Pathway G: ingestion of fluoride supplements.

Ingestion of fluoride supplements can be a major exposure pathway for some children. These supplements are prescribed to infants and children in areas that lack fluoridated public water supplies. Although several studies indicate that supplements are often prescribed inappropriately to children in fluoridated areas (Lalumandier and Rozier 1995; Pendrys and Katz 1989), we assumed that only children living in nonfluoridated areas receive supplementation. The ADA, the American Academy of Pediatric Dentistry, and the American Academy of Pediatrics recommend supplemental fluoride intake of 0.25 and 0.5 mg/day, for children 6 months to 3 years of age and children 3–6 years of age respectively, in areas with nonfluoridated water (CDC 2001). After the recommended dosing schedule for infants, exposure was limited to 6 months for infants < 1 year, starting at 6 months of age.

### Pathway H: incidental ingestion of tooth-paste.

Because > 90% of toothpaste sold in North America is fluoridated, many children are exposed to fluoride through incidental ingestion of toothpaste. Toothpastes specifically flavored for children have been linked with use of larger quantities of toothpaste, increasing the importance of this pathway (Levy 1994). The recommended concentration for fluoride ion in the United States is generally 1,000 mg/kg (ATSDR 2001; CDC 2001). The CTE and RME ingestion rates of toothpaste used to estimate EDI were the average (0.26 g toothpaste per brushing) and 90th percentile (0.77 g toothpaste per brushing) compiled from 11 studies (CDC 2001; Levy 1993, 1994). We assumed a brushing frequency of once daily for the CTE and three times daily for the RME. This pathway was excluded from the estimation of cumulative fluoride intake for infants (< 1 years of age) because several studies show that many in this age group do not have their teeth brushed (Levy et al. 1997; Tabari et al. 2000).

The EDI representing CTE and RME scenarios are calculated for each exposure pathway discussed above using Equation 1. Cumulative EDI of fluoride is estimated by adding EDI values for infants and children living in fluoridated and nonfluoridated areas using Equations 2–5. In nonfluoridated areas, fluoride concentration in drinking water was assumed to be zero; thus, intake through ingestion of drinking water was not considered. On the other hand, it was assumed that no intake via ingestion of fluoride supplements would occur in fluoridated areas.

For infants living in fluoridated areas:


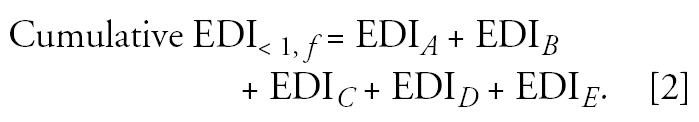


For children 3–5 years of age living in fluoridated areas:


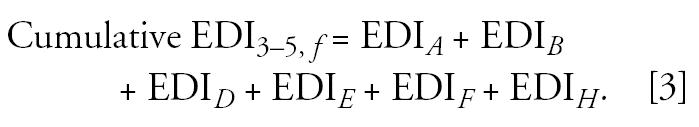


For infants living in nonfluoridated areas:


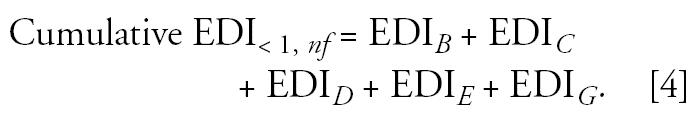


For children 3–5 years of age living in nonfluoridated areas:


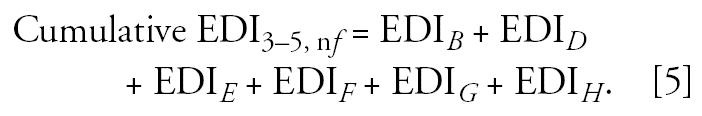


### Risk characterization.

The hazard quotient (HQ), as an estimate of the RME and CTE health risks associated with fluoride exposure via each exposure pathway, is estimated by integrating exposure and toxicity information. The sum of the HQs, the hazard index (HI), is then calculated by dividing cumulative dose (EDI) by the safe dose (RfD) using Equation 6, which represents the total fluoride intake risk:


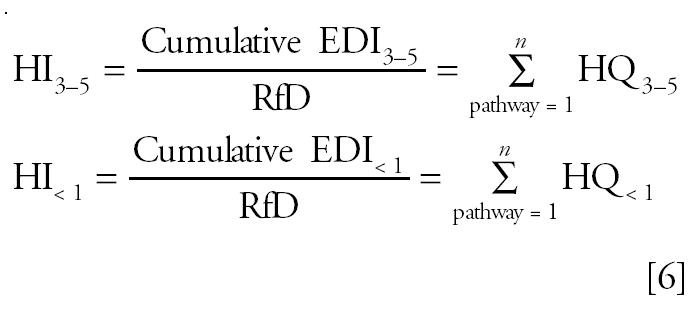


## Results

**Figure 1:**
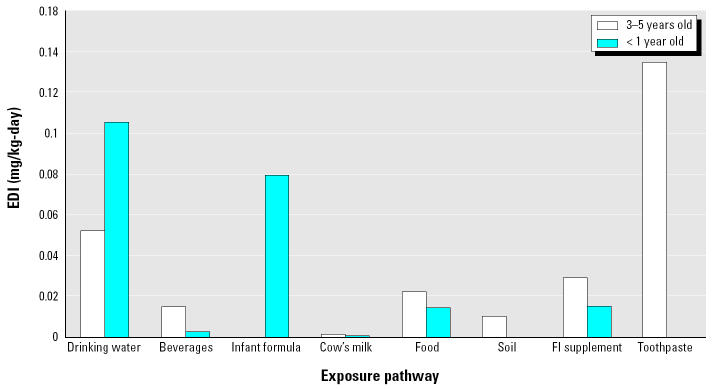
RME daily intake estimates for each exposure pathway.

Numerical results of the CTE and RME EDI estimates for each pathway and for each age group are shown in Table 2. Figure 1 depicts the RME EDI estimates for infants and children. For infants, although drinking water (52%) and infant formula (39%) are the two most significant sources contributing to cumulative daily fluoride intake in fluoridated areas, infant formula (71%), fluoride supplements (13.4%), and food (12.9%) are the sources of importance in nonfluoridated areas. For children, toothpaste (57%), drinking water (22%), and food (9%) in fluoridated areas and tooth-paste (63%), fluoride supplements (14%), and food (10%) in nonfluoridated areas contribute significantly to the cumulative daily intake under the RME conditions.

**Figure 2:**
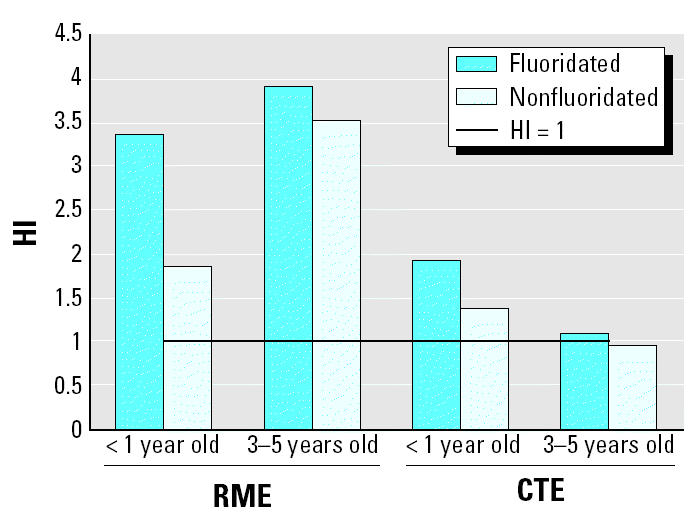
HI estimates for each exposure scenario.

Tables 3 and 4 show the HQ and HI values estimated for infants and children for each exposure scenario. Table 4 also documents the estimates of cumulative EDI applicable to each exposure group living in fluoridated or nonfluoridated communities. All of the HI values are around unity for all CTE estimates, slightly elevated for infants living in fluoridated areas. On the other hand, all HI estimates for the RME scenario are greater than unity, indicating that cumulative daily fluoride intake is greater than the safe dose level established by the EPA for fluoride. Therefore, there may be a segment of both populations who are at risk of developing fluorosis due to exposure to fluoride via exposure pathways studied in this analysis, under the specified RME conditions. Figure 2 illustrates the HI estimates for each exposure group in relation to the acceptable standard of unity for HI. It is interesting to note that all exposure-pathway–specific HQ values are less than or around unity for infants and children under CME conditions, as shown in Table 3. However, fluoridated drinking water ingestion and consumption of infant formula for infants and incidental ingestion of toothpaste by children are associated with HQ values slightly greater than unity under the RME conditions, which result in HI estimates greater than unity.

The cumulative daily fluoride intake in fluoridated areas was estimated at 0.20 and 0.11 mg/kg-day for RME and CTE scenarios, respectively, for infants. On the other hand, the RME and CTE estimates for children were 0.23 and 0.06 mg/kg-day, respectively (Table 4). In areas where municipal water is not fluoridated, our RME and CTE estimates for cumulative daily average intake were, respectively, 0.11 and 0.08 mg/kg-day for infants and 0.21 and 0.06 mg/kg-day for children. For infants, cumulative fluoride intake is all due to dietary sources in fluoridated areas. In non-fluoridated areas, dietary intake constitutes about 87% (0.1 mg/kg-day) and 90% (0.07 mg/kg-day) of the cumulative intake for infants and about 18% (0.04 mg/kg-day) and 43% (0.02 mg/kg-day) of the cumulative daily intake for children under RME and CTE scenarios, respectively. In fluoridated areas, dietary intake constituted 38% (0.09 mg/kg-day) and 73% (0.05 mg/kg-day) of the cumulative intake for children for RME and CTE scenarios, respectively. These results demonstrate that total fluoride exposure is due mainly to dietary sources for infants; however, non-dietary sources (e.g., fluoride supplements, toothpaste) gain importance for children’s exposure to fluoride. The average optimum dietary fluoride intake by children living in fluoridated communities is found to be close to 0.05 mg/kg-day (range, 0.02–0.1 mg/kg-day; Institute of Medicine 1999). The Institute of Medicine recently established a tolerable upper intake level of 0.1 mg/kg-day for infants, toddlers, and children ≤8 years of age, based on the lowest observed adverse effect level for moderate fluorosis, using dietary fluoride intake data (Institute of Medicine 1999). All of our dietary intake estimates fall within the range of 0.02–0.1 mg/kg-day, except for infants living in fluoridated areas under the RME scenario, primarily due to ingestion of water. Levy et al. (2001) also found that for children < 12 months of age, drinking water was a primary source of fluoride intake.

## Discussion

Several studies published in the literature have estimated total daily fluoride intakes from dietary sources and toothpaste ingestion. Pendrys and Stamm (1990) estimated fluoride intake from diet (water and beverages), supplements, and toothpaste to be 0.07 (range, 0.04–0.2) and 0.08 (range, 0.05–0.21) mg/kg-day for children 2 years of age from fluoridated and nonfluoridated communities, respectively. These EDI estimates are similar to our estimates for 3- to 5-year-old children, with the mean of 0.06 mg/kg-day in both fluoridated and non-fluoridated water areas. It is interesting to note that the high-end estimates reach approximately 0.2 mg/kg-day in both analyses. Levy et al. (2001) estimated daily fluoride intake from water (including beverages), toothpaste, and fluoride supplements from birth to 36 months of age as part of a longitudinal study in Iowa. The estimated mean intakes were 0.06 mg/kg for the 3- to 12-month-old group and 0.043 mg/kg-day for the 20- to 36-month-old group. The 90th percentile values were 0.12 and 0.08 mg/kg-day for infants (3–12 months of age) and 3-year-old children, respectively. On the other hand, the maximum fluoride intake estimates were significantly higher, amounting to 0.2 mg/kg-day for 3-year-old children and 0.9 mg/kg-day for infants. We estimated average intake levels of 0.05 and 0.06 mg/kg-day for < 1- and 3- to 5-year-old age groups in our analysis for combined fluoride sources from water, beverage, fluoride supplement, and toothpaste, which agree with the estimates of Levy et al. (2001). Our RME EDI estimates for these four exposure pathways were 0.12 and 0.23 mg/kg-day for infants and children, respectively. Although the infant estimate agrees with the reported 90th percentile value, our RME for children is higher (close to maximum), potentially because, although we consider older children, Levy et al. (2001) limits the maximum age studied to 3 years. In addition, Levy et al. (2001) did not include intake of prepackaged beverages such as fruit juice and soda, and the amount of toothpaste used and proportion ingested was estimated by parents, both of which may have led to underestimation of fluoride intake. Jackson et al. (2002) recently estimated average daily dietary intake of fluoride from food (including milk) and beverages using a food questionnaire and USDA intake rates for 3- to 5-year-old children living in fluoridated and nonfluoridated towns in Indiana. The children from the fluoridated town had an average fluoride daily intake of 0.033 mg/kg-day and a maximum intake of 0.062 mg/kg-day. On the other hand, children from nonfluoridated communities had an average of 0.028 mg/kg-day and a maximum intake of 0.058 mg/kg-day. Our EDI estimates for these pathways (milk, food, beverages) for the 3- to 5-year-old exposure group are slightly lower, with 0.04 and 0.02 mg/kg-day for RME and CTE scenarios, respectively. This may be because our estimates for fluoride intake through milk, beverages, and food do not differentiate whether these sources come from fluoridated or nonfluoridated areas.

Prevention of dental caries, as established by numerous epidemiologic studies, has been such a dramatic public health achievement that the U.S. Public Health Service has set a goal of increasing the percentage of the U.S. population being served by a fluoridated supply to 75%, in its Healthy People 2010 initiative (U.S. DHHS 2000b). However, the findings of this health risk assessment study support concerns that a segment of the infant and child population in the United States may be exposed to amounts of fluoride greater than the optimum level for caries prevention. We found that when only dietary exposure pathways are considered, the EDI varies from 0.02 to 0.1 mg/kg-day in nonfluoridated communities, which is within the optimum range (Institute of Medicine 1999). However, in fluoridated communities, this range was 0.05–0.2 mg/kg-day, with drinking water and infant formula being the primary contributors. When nondietary sources were also considered, the cumulative EDI values significantly increased for children, whereas there was a negligible difference between the dietary exposure and cumulative exposure for infants. For the 3- to 5-year-old age group, the use of fluoride supplements and, especially, inadvertent ingestion of toothpaste containing fluoride significantly increased the total fluoride intake by 2- to 6-fold under the RME scenario. However, drinking water, food, fluoride supplements, and toothpaste contributed in similar percentages (21–27%) to the cumulative EDI under the CTE scenario for children living in fluoridated communities.

Analysis of uncertainty is an essential component of risk assessment. In this study, we used a single point value for each of the exposure parameters. However, fluoride concentrations in drinking water, beverages and fruit juices, and various food items are known to vary greatly (Jackson et al. 2002). Studies measuring fluoride concentration in beverages do not track products to their source to verify whether they were produced with fluoridated water (Heilman et al. 1999; Jackson et al. 2002; Levy 1994; Pang et al. 1992). A child consuming only beverages prepared with non-fluoridated water would have a lower fluoride intake. Children brushing more or less often obviously increase or decrease their risk of swallowing toothpaste. Children using mouth rinses and gels and specially flavored tooth-paste may especially be increasing their fluoride intake. Because of the availability of scant data on intake rates of bottled water, this source was not considered. The increased reliance on bottled water as the primary drinking source among the U.S. population may change the dynamics of fluoride intake among the children, especially given the fact that many bottled water products do not contain any fluoride. That is why it is paramount to continuously track the prevalence of dental caries and fluorosis at specific life stages to determine trends and to apportion the total intake into each source. Tea leaves contain high levels of fluoride, and brewed tea concentrations can range from 1 to 6 mg/L (Institute of Medicine 1999; Pang et al. 1992). Children growing up in ethnic communities with frequent tea consumption may have increased high intake of fluoride (Cao et al. 1997; Jin et al. 2000). An epidemiologic investigation carried out in Mexico showed that boiling water doubled fluoride concentrations found in nonboiled water (Grimaldo et al. 1995). Thus, food or infant formula prepared with boiled water may result in increased fluoride intake through diet. The uncertainty associated with concentrations of fluoride in drinking water, drinking water ingestion rate, consumption rates of beverages, cow’s milk, food, and fluoride supplement dosage can be classified as relatively low because these estimates emanate from national-scale studies conducted by the U.S. EPA and USDA. The uncertainty for the rest of the exposure parameters fall into “high” or “medium to high” categories. Fluoride concentrations in various exposure media (e.g., beverages, cow’s milk, infant formula, food, soil) and incidental toothpaste ingestion rate are especially uncertain. Therefore, the uncertainty in the overall intake and risk estimates can be described as “medium” at best, most likely as “medium to high.”

The HI, which considers all exposure pathways applicable for a given exposure group, was greater than unity in all cases under the RME conditions and was within acceptable ranges in all cases except for infants living in fluoridated areas under the CTE conditions. Therefore, it is likely that some infants and children receive fluoride levels in excess of those “likely to be without appreciable deleterious effects” (U.S. EPA 2003) and are at risk for fluorosis. The findings of this study confirm the importance of considering all potentially applicable exposure pathways in estimating cumulative daily fluoride dose for scientifically sound decision making in fluorosis risk management. Although the EDI associated with the ingestion of drinking water pathway (RME: 0.05 mg/kg-day, children; 0.1 mg/kg-day, infants; CTE: 0.02 mg/kg-day, children; 0.04 mg/kg-day, infants) does not exceed the optimum fluoride range in fluoridated areas by itself, the cumulative intake exceeds the optimum range when other pathways are considered. Therefore, one approach could be implementation of measures to reduce fluoride intake from sources other than water in communities where tap water is fluoridated. The risk management for fluorosis in these communities could focus on preparation of infant formula for infants and ingestion of toothpaste for children. This finding emphasizes the significance of educating parents and child-care specialists about fluorosis risk by public health practitioners, physicians, and dentists. The fluorosis risk can easily be reduced by supervising children while brushing and by preparing infant formula with nonfluoridated water or purchase of infant formula constituted without fluoride. A significant role in fluorosis risk management is also assumed by the public health, medical, and dental professionals by accurately diagnosing fluoride needs of children by inquiring about all sources that are associated with fluoride exposure on a case-by-case basis and making informed and educated decisions about fluoride supplement prescription unique to each child.

On the other hand, a significant finding of our analysis is that, for both age groups living in nonfluoridated areas, although under the CTE scenario the cumulative intake is within the optimum range (0.06 mg/kg-day for children, 0.08 mg/day for infants), under the RME scenario the cumulative intake estimates are higher (0.21 mg/kg-day for children, 0.11 mg/kg-day for infants), exceeding the optimum range. This raises questions about the continued need for fluoridation in the U.S. municipal water supply to protect against the risk of fluorosis. However, given the uncertainties inherent in this analysis, it is not possible to be conclusive. Further research with carefully designed epidemiologic studies with enough statistical power and strong exposure assessment component is essential and warranted to answer critical questions about the necessity of fluoridation in the presence of changes in dietary behavior of children and multiple sources of fluoride currently contributing total intake. Cost–benefit analysis for fluoride should be a component of such studies. In addition, future studies should lead to collection of detailed exposure data for each exposure pathway so that more robust probabilistic risk assessment techniques, as opposed to point estimates of intake/risk presented here, can be applied to obtain distribution of fluoride intake/risk among children with quantitative measures of uncertainty.

## Figures and Tables

**Table 1:** Summary of exposure parameters used in the calculation of estimated daily fluoride intake.

Exposure pathway	Fluoride concentration	CTE intake rate*^a^*	RME intake rate
A. Drinking water*^b^*	1 mg/L	< 1 year old: 0.34 L/day	< 1 year old: 0.88 L/day
		1–10 years old: 0.4 L/day (U.S. EPA 2002)	1–10 years old: 0.9 L/day (U.S. EPA 2002)
B. Beverages*^c^*	0.76 mg/L (Pang et al. 1992)	< 1 year old: 19 g/day	< 1 year old: 23.75 g/day
		3–5 years old: 269 g/day (U.S. EPA 2002)	3–5 years old: 336.25 g/day (U.S. EPA 2002)
C. Infant formula	0.65 mg/kg (ATSDR 2001)	198 mL/feeding, 4.4 feedings/day (Behrman and Vaughn 2000)	214 mL/feeding, 4.8 feedings/day (U.S. EPA 2002Behrman and Vaughn 2000)
D. Cow’s milk*^c^*	0.041 mg/kg (Dabeka and McKenzie 1987)	< 1 year old: 61 g/day	< 1 year old: 76.25 g/day
		3–5 years old: 335 g/day (U.S. EPA 2002)	3–5 years old: 418.75 g/day (U.S. EPA 2002)
E. Food*^b^*	< 1 year old: 0.262 mg/kg	< 1 year old: 223.6 g/day	< 1 year old: 612.7 g/day
	3–5 years old: 0.290 mg/kg (Dabeka and McKenzie 1995)	3–5 years old: 691.9 g/day (U.S. EPA 2002)	3–5 years old: 1,312.5 g/day (U.S. EPA 2002)
F. Soil*^d^*	430 mg/kg (ATSDR 2001)	0.1 g/day (U.S. EPA 2002)	0.4 g/day (U.S. EPA 2002)
G. Fluoride supplements		6 months to 10 years of age: 0.25 mg/day NaF	6 months to 3 years old: 0.25 mg/day NaF
		3–6 years old: 0.5 mg/day NaF (CDC 2001)	3–6 years old: 0.5 mg/day NaF (CDC 2001)
H. Toothpaste	1,000 mg/kg (ATSDR 2001)	3–5 years old: 0.26 g/brushing (Levy 1993)	3–5 years old: 0.77 g/brushing (Levy 1993)
		1 brushing/day	3 brushings/day

a Recommended mean intake rate as a combined estimate for males and females was used in all cases in the CTE scenario.

b For drinking water and food consumption, 90th percentile of recommended intake rate was used in the RME scenario.

c For consumption of beverages and cow’s milk, 25% more consumption than the mean was assumed in the estimation of RME daily intake.

d For incidental ingestion of soil by children, upper percentile ingestion rate was used in the RME scenario.

**Table 2:** EDI (mg/kg-day) estimates for CTE and RME exposure scenarios.

	CTE		RME	
Exposure pathway	< 1 year old	3–5 years old	< 1 year old	3–5 years old
A. Fluoridated drinking water	0.04	0.023	0.10	0.052
B. Beverages	0.0017	0.012	0.021	0.015
C. Infant formula	0.067	NA	0.079	NA
D. Cow’s milk	0.0003	0.0008	0.00037	0.001
E. Food	0.0052	0.012	0.014	0.022
F. Soil	NA	0.0025	NA	0.01
G. Fluoride supplements	0.0074	0.014	0.015	0.029
H. Toothpaste	NA	0.015	NA	0.13

NA, exposure pathways assumed to be not applicable.

**Table 3:** The CTE and RME HQ estimates for individual exposure pathways.

	CTE		RME	
Exposure pathway	< 1 year old	3–5 years old	< 1 year old	3–5 years old
A. Fluoridated drinking water	0.7	0.4	1.7	0.9
B. Beverages	0.03	0.2	0.04	0.2
C. Infant formula	1.1	NA	1.3	NA
D. Cow’s milk	0.005	0.01	0.006	0.02
E. Food	0.09	0.2	0.2	0.4
F. Soil	NA	0.04	NA	0.2
G. Fluoride supplements	0.1	0.2	0.2	0.5
H. Toothpaste	NA	0.2	NA	2.2

NA, exposure pathways assumed to be not applicable.

**Table 4:** HIs and cumulative EDIs (mg/kg-day) for exposure scenarios of concern.

	HI		Cumulative EDI	
Exposure scenario	CTE	RME	CTE	RME
Fluoridated area				
< 1 year old	1.9	3.3	0.11	0.20
3–5 years old	1.1	3.9	0.06	0.23
Nonfluoridated area				
< 1 year old	1.4	1.8	0.08	0.11
3–5 years old	0.9	3.5	0.06	0.21
